# Supplementary material for: Decoupled electrolysis for hydrogen production and hydrazine oxidation via high-capacity and stable pre-protonated vanadium hexacyanoferrate
Source: Nat Commun. 2024 Feb 13;15:1339. doi: 10.1038/s41467-024-45321-z (PMC10864379; doi:10.1038/s41467-024-45321-z)
Supplement: Supplementary file 1 — Supplementary Information [file 41467_2024_45321_MOESM1_ESM.pdf]

Supplementary Information

Decoupled electrolysis for hydrogen production and hydrazine oxidation via high-capacity and stable pre-protonated vanadium hexacyanoferrate

Fei Lv[1], Jiazhe Wu[1], Xuan Liu[1], Zhihao Zheng[1], Lixia Pan[1], Xuewen Zheng[1], Liejin Guo[1], Yubin Chen[1]*

[1]International Research Center for Renewable Energy, State Key Laboratory of Multiphase Flow in Power Engineering, Xi'an Jiaotong University, Shaanxi 710049, China

[*]Corresponding author.

E-mail address: ybchen@mail.xjtu.edu.cn

TABLE OF CONTENTS

Supplementary Figs. 1-23 Pages S2-S13

Supplementary Table 1 Page S13

Supplementary References Page S14

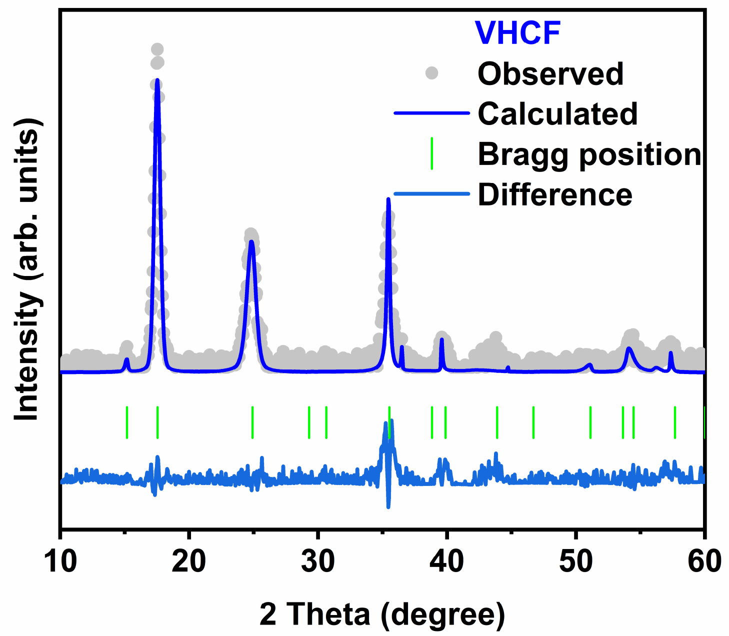

Supplementary Fig. 1 The XRD pattern and its Rietveld refinement profile of VHCF.

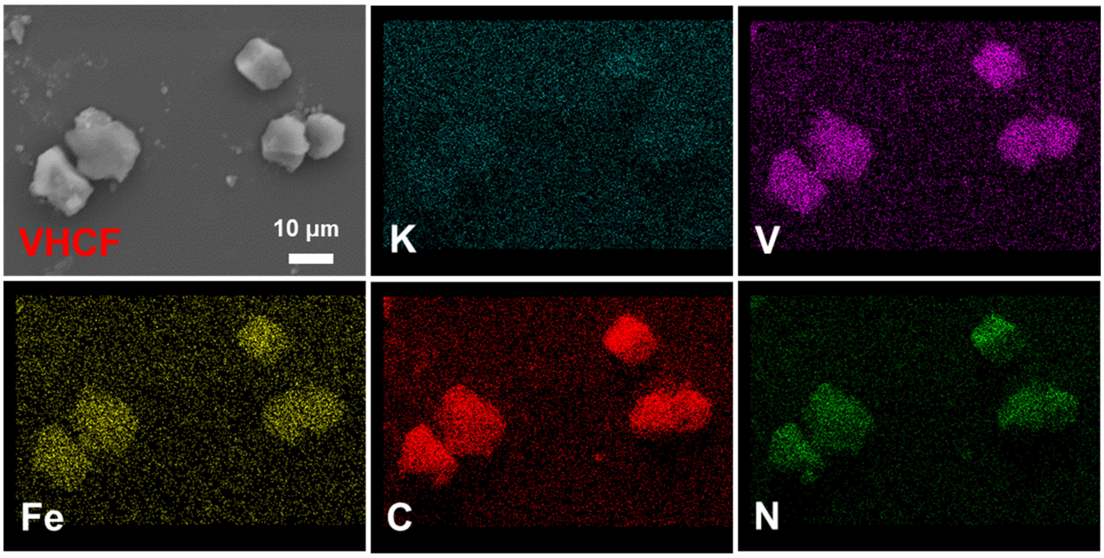

Supplementary Fig. 2 The EDS mapping of the VHCF powder.

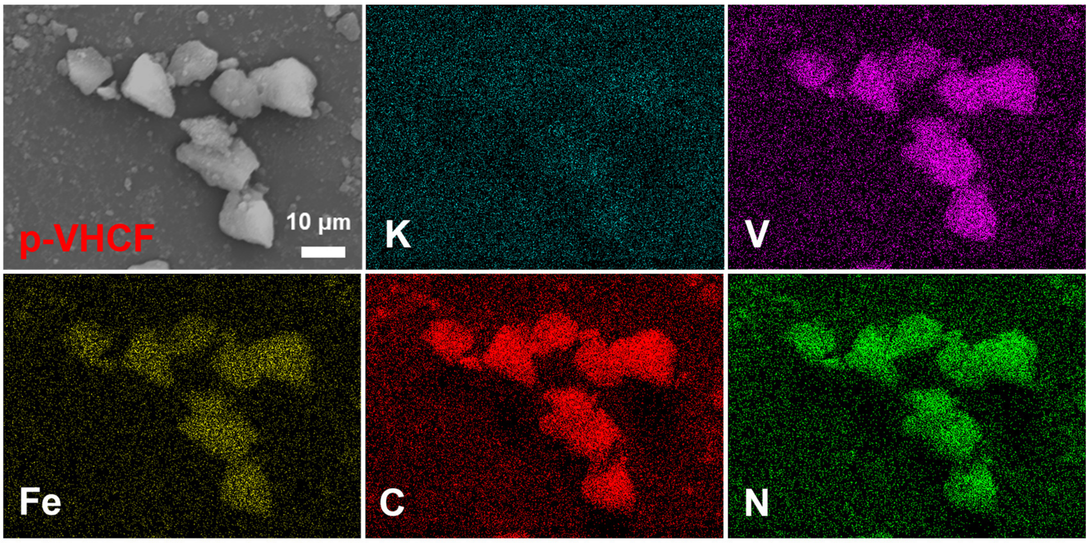

Supplementary Fig. 3 The EDS mapping of the p-VHCF powder.

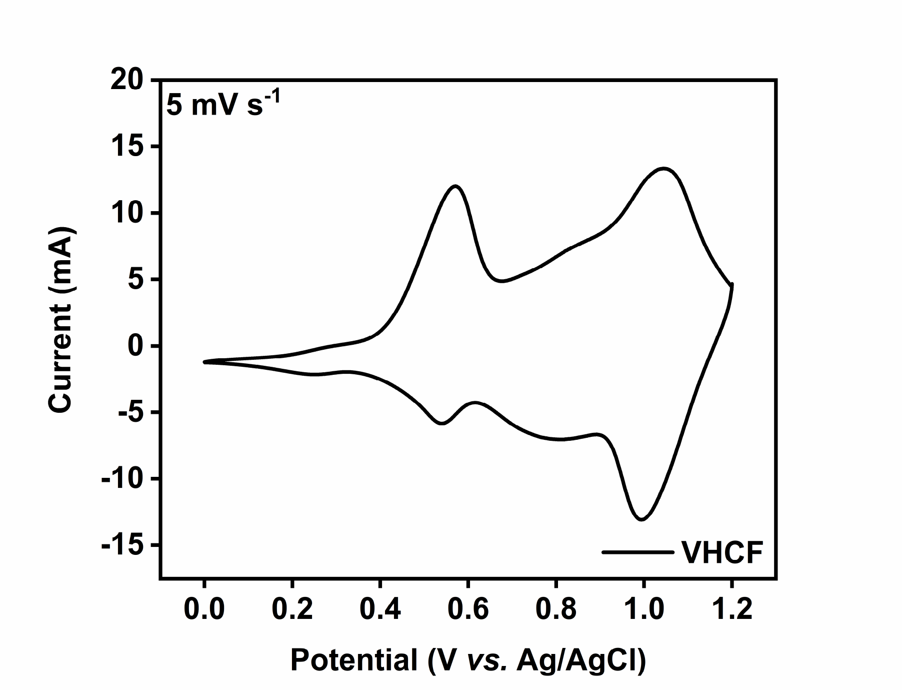

Supplementary Fig. 4 CV curve of the VHCF electrode at a scan rate of 5 mV s^{-1}.

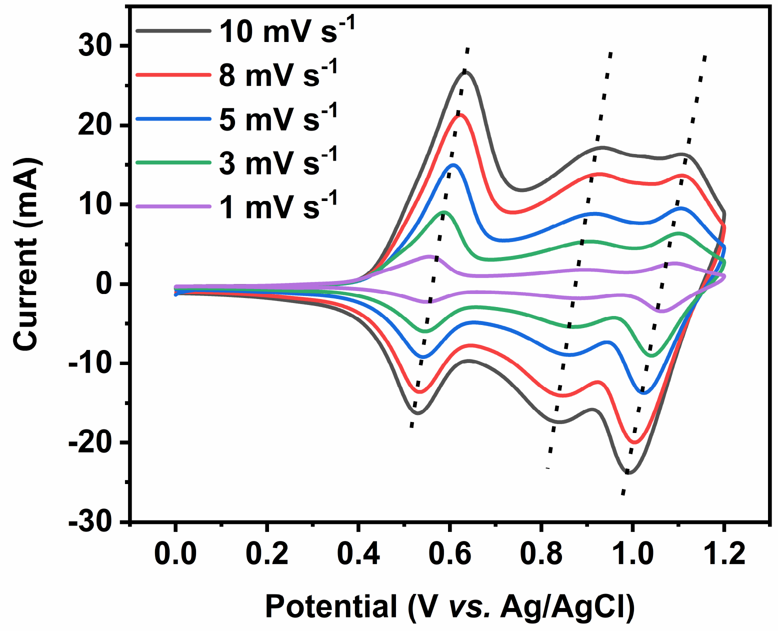

Supplementary Fig. 5 CV curves of the p-VHCF electrode under different scan rates.

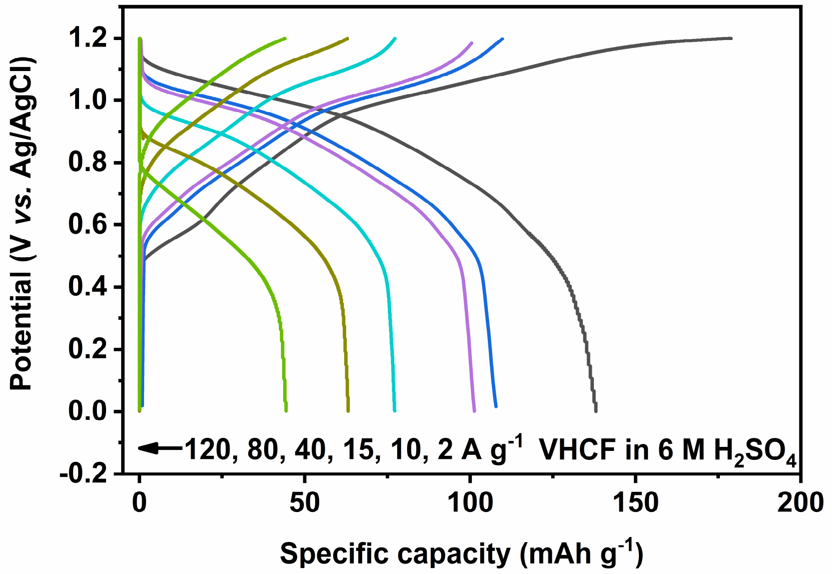

Supplementary Fig. 6 Rate performance of the VHCF electrode at various current densities in 6 M H_2SO_4 electrolyte.

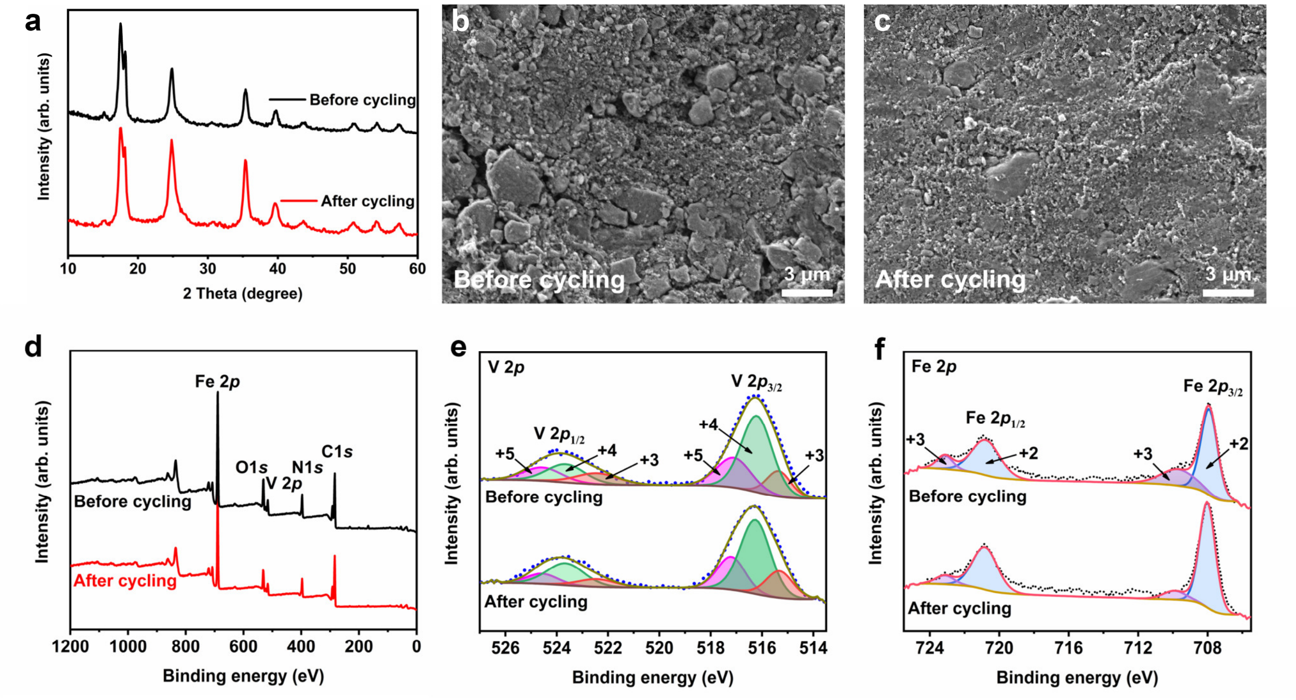

Supplementary Fig. 7 Structure and morphology of the p-VHCF electrode before and after the 6000 cycles. (a) XRD patterns (measured with p-VHCF film electrode). (b, c) SEM images before and after long cycling. (d) XPS survey spectra. (e, f) High-resolution XPS spectra of V 2p and Fe 2p.

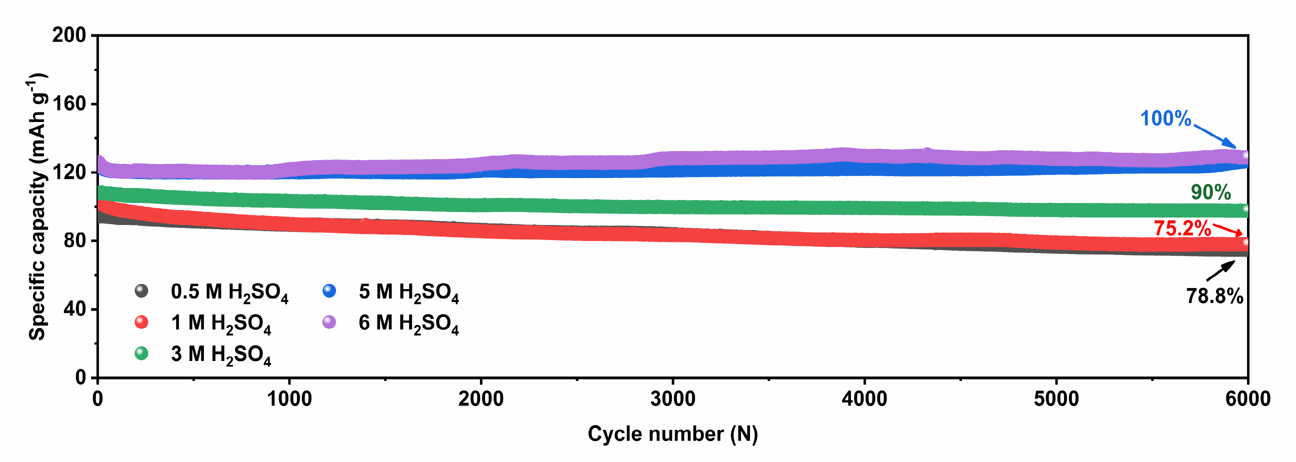

Supplementary Fig. 8 Cycle performances of p-VHCF electrode at 10 A g^{-1} for 6000 cycles in 0.5, 1, 3, 5 and 6 M H_2SO_4.

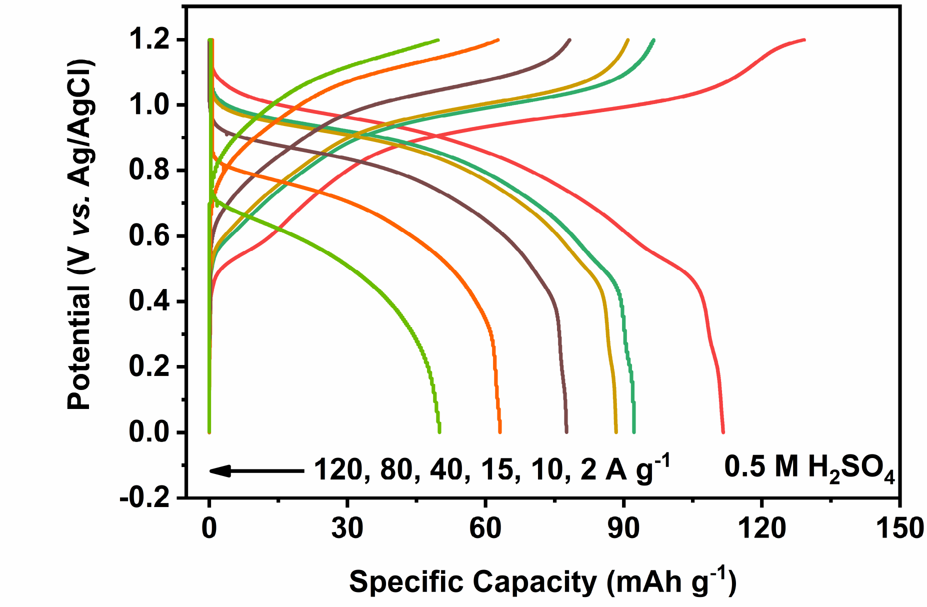

Supplementary Fig. 9 Rate performance of the p-VHCF electrode at varied current density in 0.5 M H_2SO_4 electrolyte.

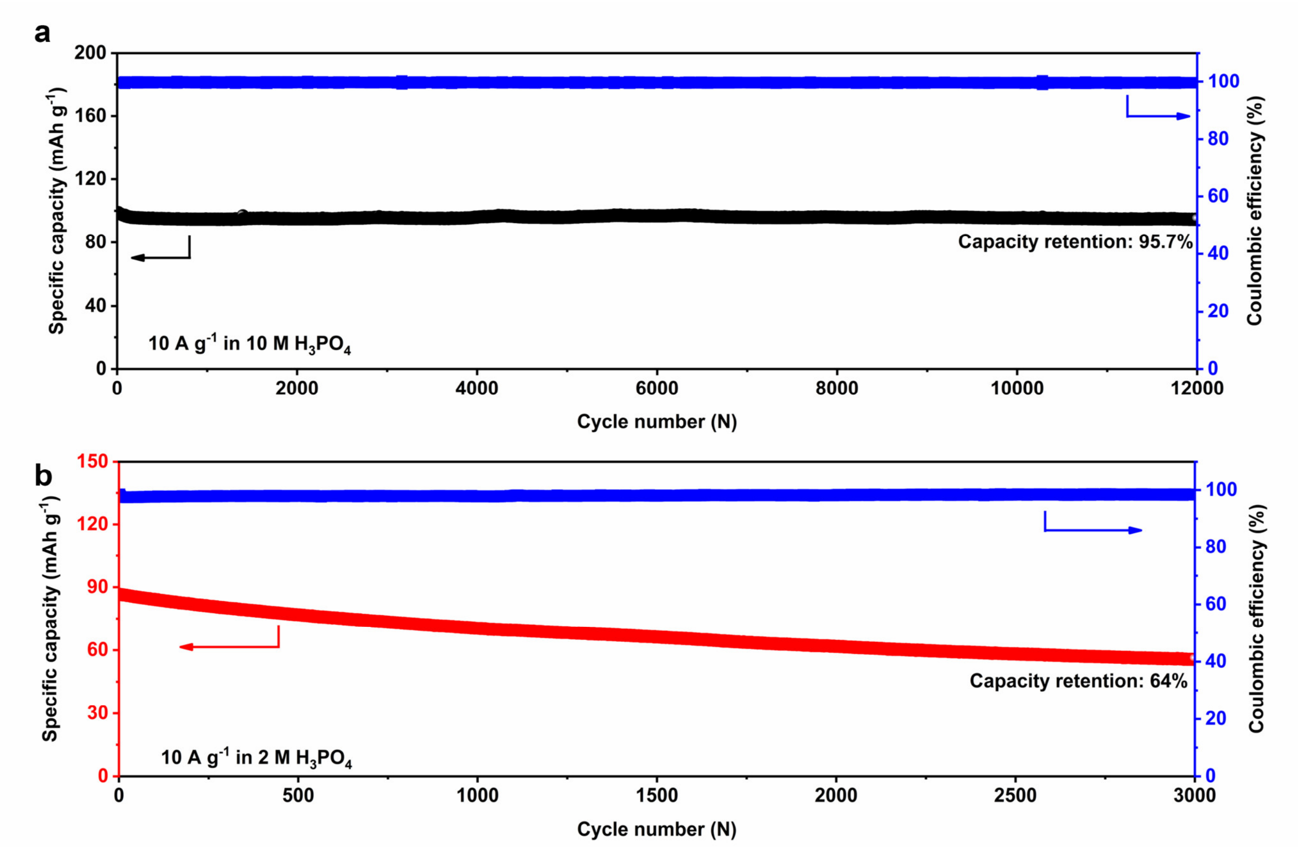

Supplementary Fig. 10 Cycling performance of the p-VHCF electrode in (a) 10 M and (b) 2 M H_3PO_4 electrolyte at 10 A g^{-1}.

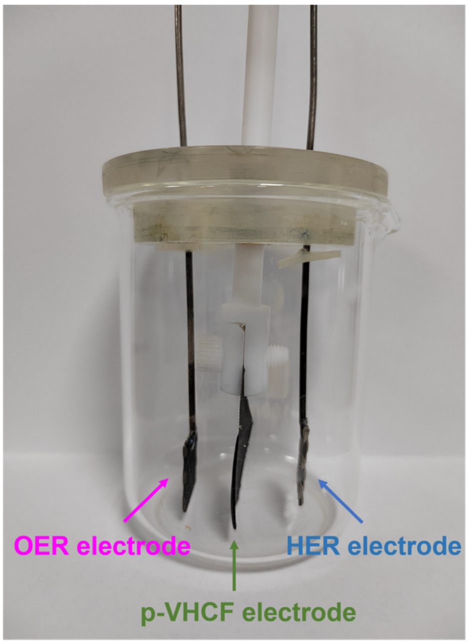

Supplementary Fig. 11 The image of the decoupled acid water electrolyzer.

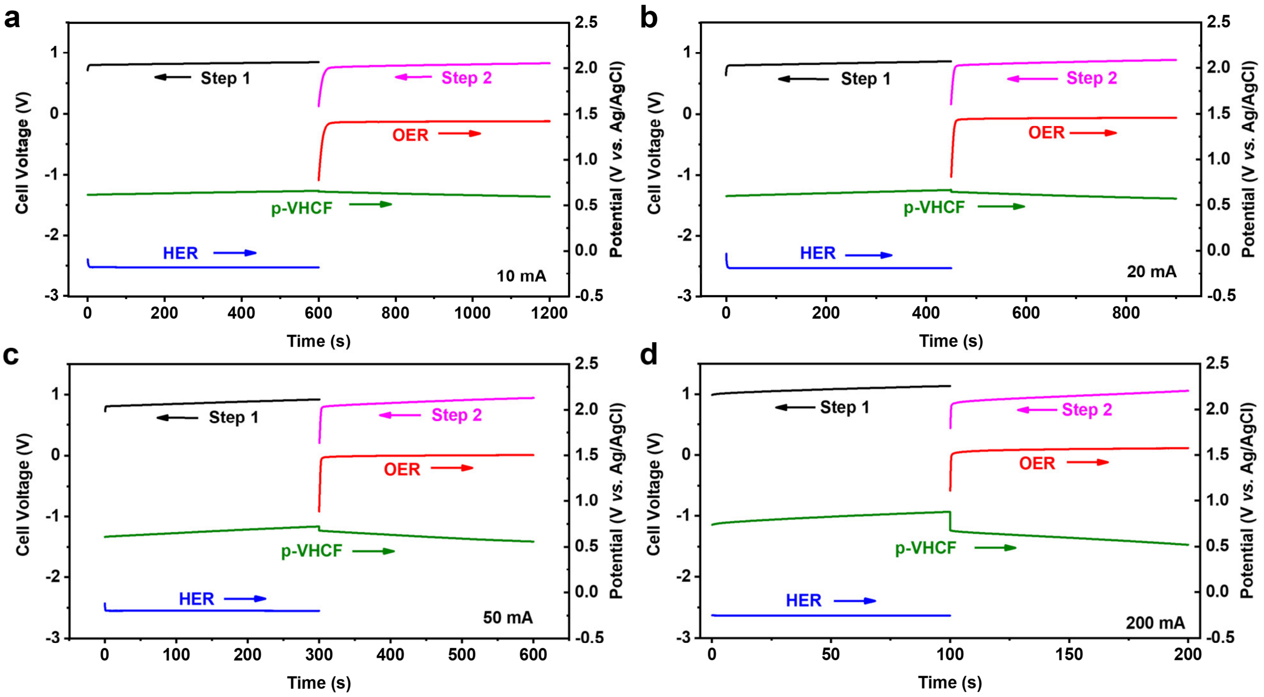

Supplementary Fig. 12 Performance of the decoupled water electrolysis under different currents. (a) 10 mA. (b) 20 mA. (c) 50 mA, and (d) 200 mA.

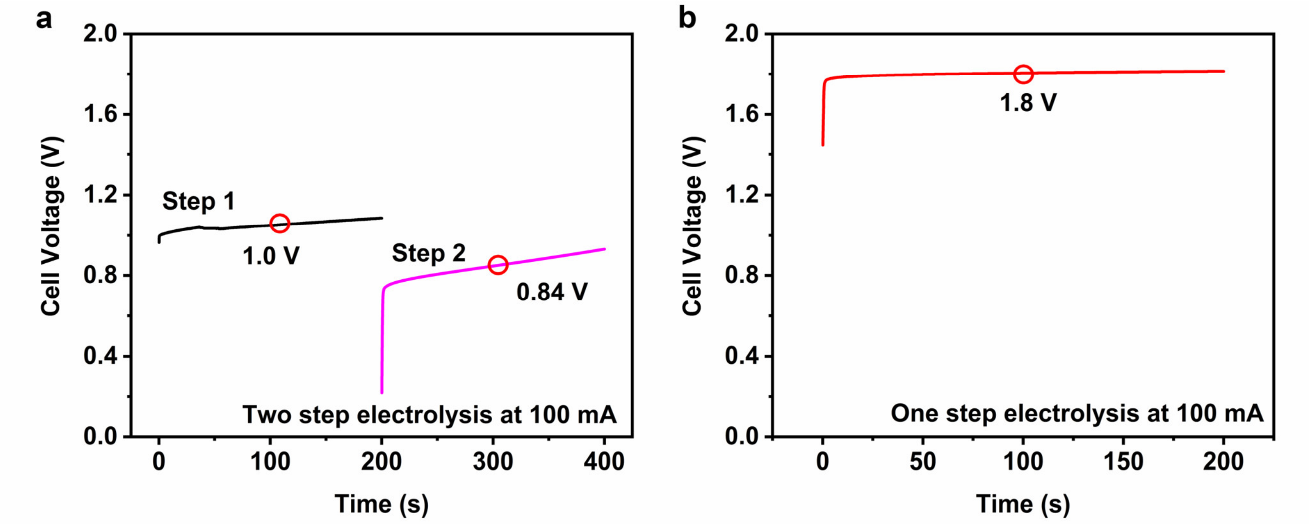

Supplementary Fig. 13 Voltage comparison at 100 mA between (a) two-step decoupled electrolysis and (b) one-step direct electrolysis without the membrane.

As shown in **Supplementary Fig. 13a**, the two steps display a total cell voltage of 1.84 V (1.0 + 0.84 V). The chronopotentiometry curve of one-step electrolysis with the same HER/OER electrodes is shown in **Supplementary Fig. 13b**, where the cell exhibits a voltage of 1.8 V with the applied current of 100 mA. Therefore, the efficiency of the decoupled cell was 97.8% (1.8/1.84) compared to the corresponding one-step system.

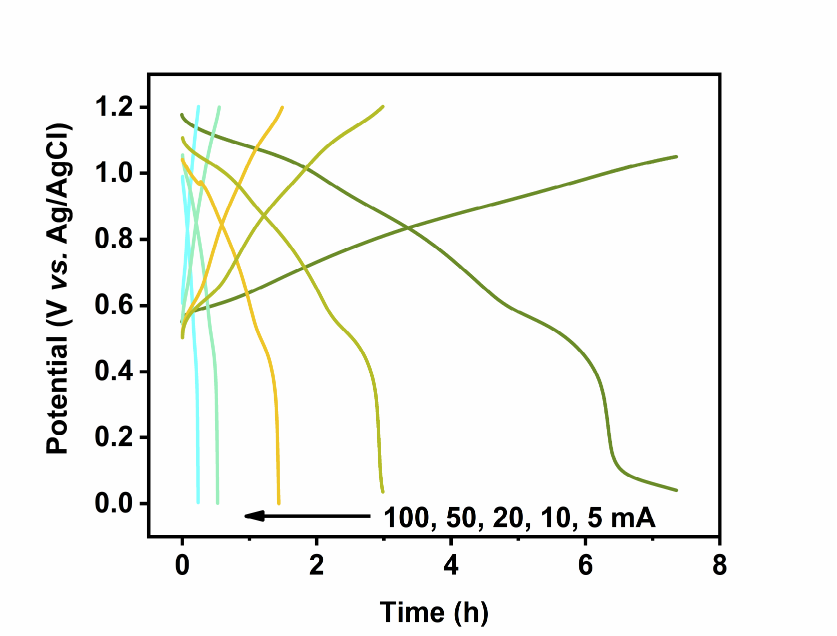

Supplementary Fig. 14 The potential of the p-VHCF redox mediator as a function of time at different currents (the mass loading of the p-VHCF is 60 mg cm^{-2}).

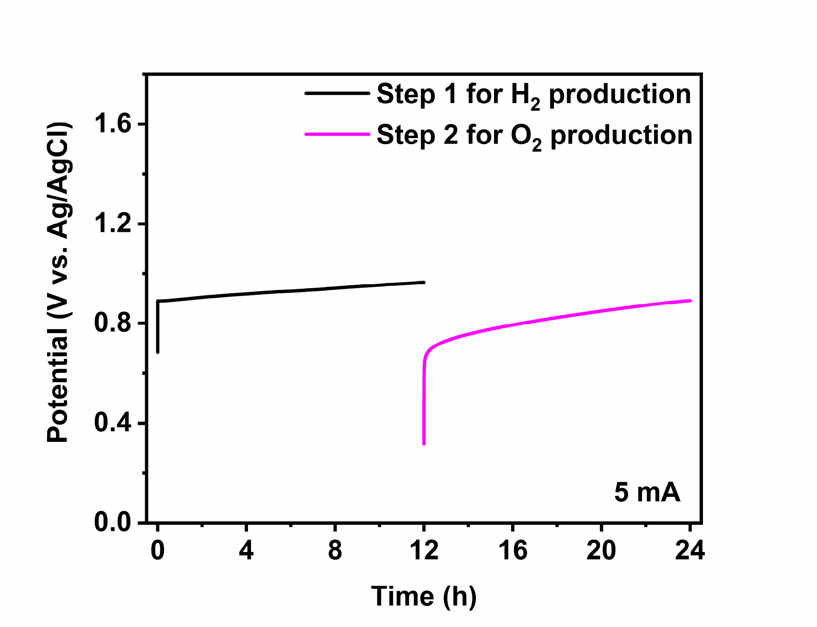

Supplementary Fig. 15 Chronopotentiometry curve of the decoupled electrolyzer with a step-time of 12 h.

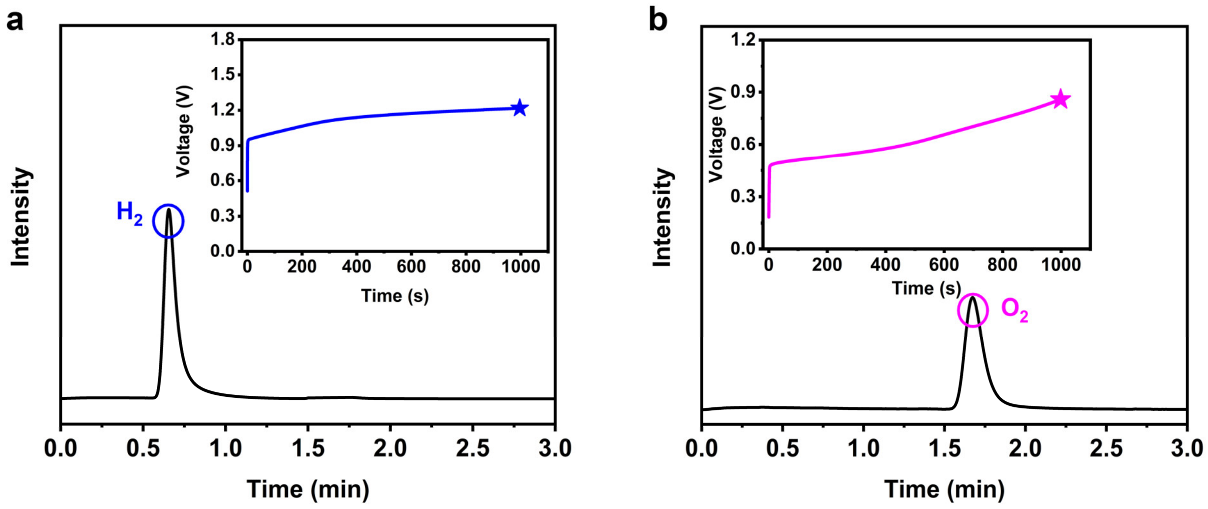

Supplementary Fig. 16 Gas chromatography data for (a) HER and (b) OER at 20 mA. Inset: corresponding chronopotentiometry curves (cell voltage vs time), where the asterisk represents the sampling time.

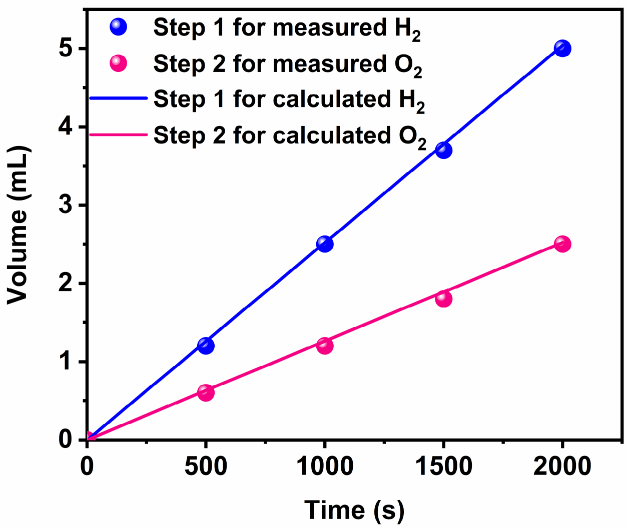

Supplementary Fig. 17 Practical amounts of obtained H_2 and O_2 at a current of 20 mA with each HER and OER step for 2000 s.

A typical drainage method was employed to quantify the H_2/O_2 production with an applied current of 20 mA for 2000 s. The theoretical values are 5.04 mL and 2.52 mL for H_2 and O_2. The actual production amounts of H_2 and O_2 are 5 mL and 2.5 mL, respectively (**Supplementary Fig. 17**). Therefore, the Faradaic efficiency of the H_2 production step is 99.2% (5/5.04) and the Faradaic efficiency of O_2 production step is 99.2% (2.5/2.52).

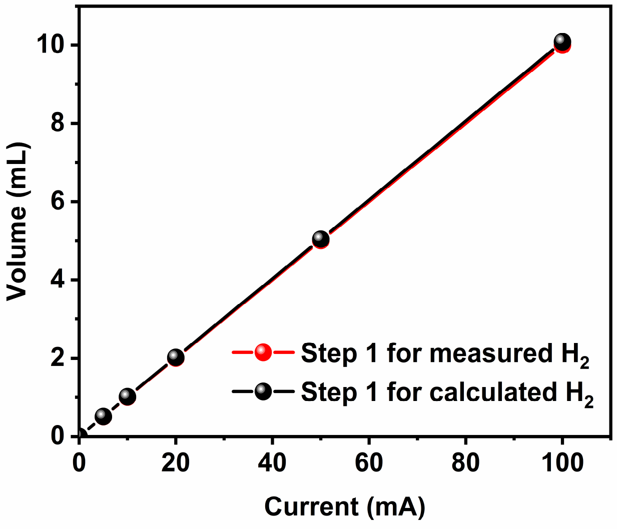

Supplementary Fig. 18 Hydrogen evolution as a function of current with all step time of 800 s.

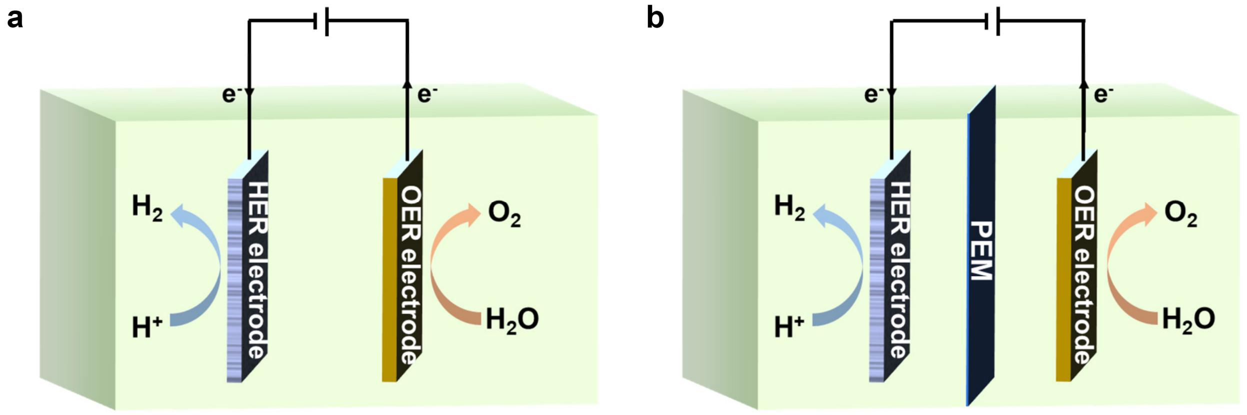

Supplementary Fig. 19 Illustration of (a) one-step water electrolysis without membrane and (b) one-step water electrolysis with the proton exchange membrane.

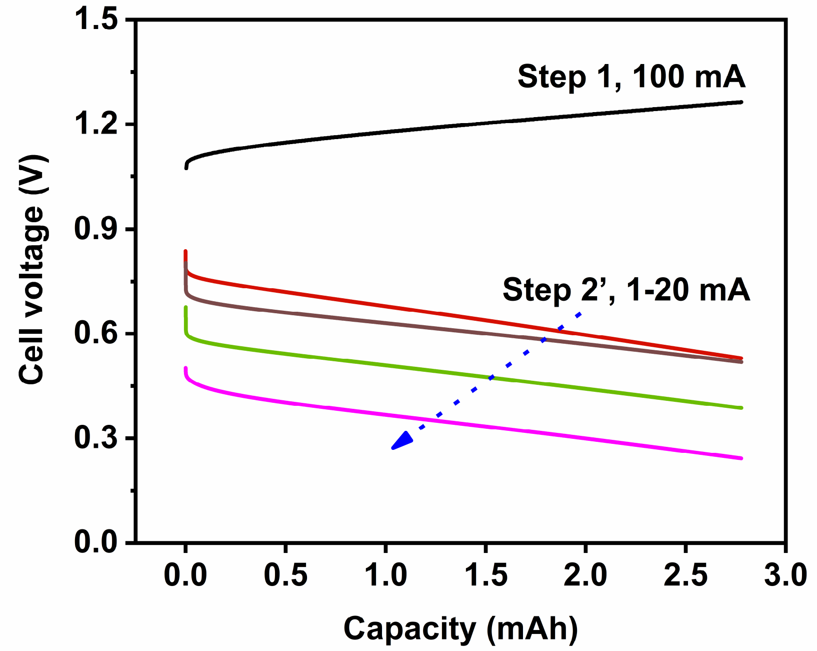

Supplementary Fig. 20 Rate performance of the p-VHCF-N_2H_4 liquid battery.

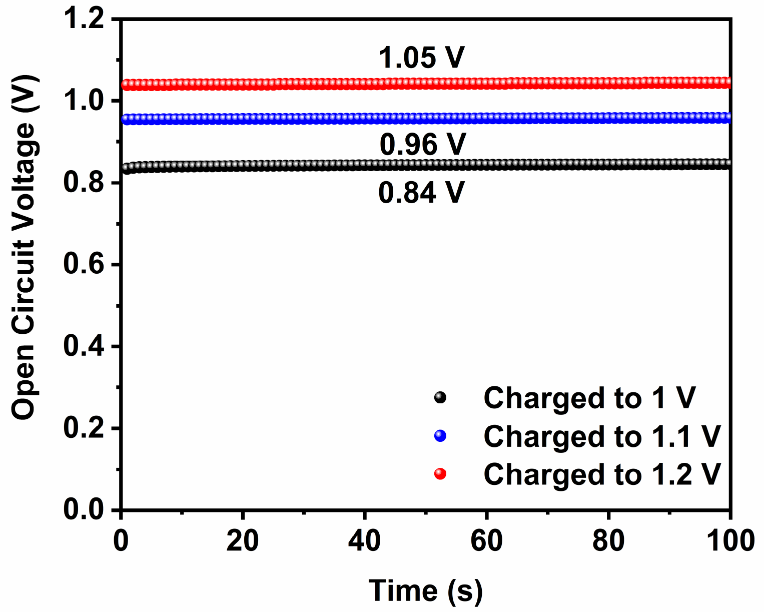

Supplementary Fig. 21 The open-circuit voltage of the p-VHCF-N_2H_4 liquid battery in Step 2' after p-VHCF electrode was charged to different potentials.

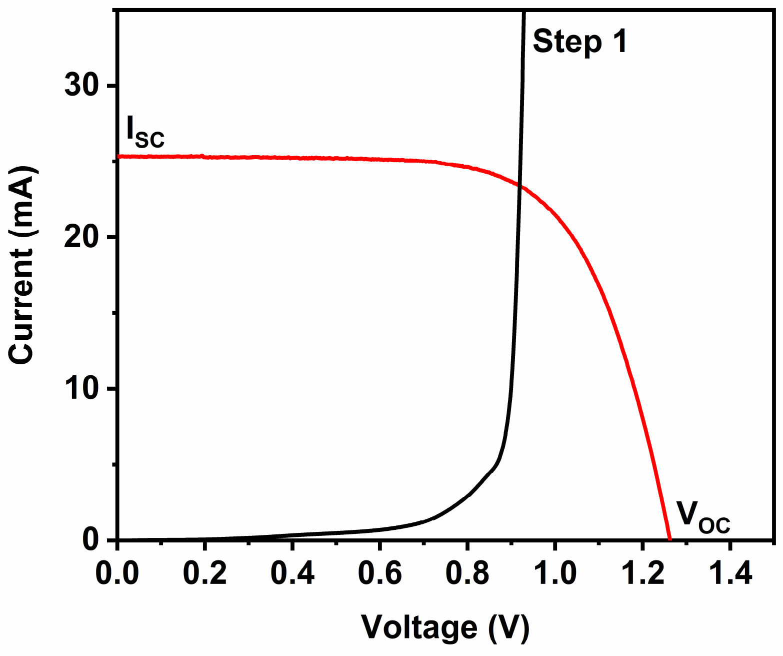

Supplementary Fig. 22 LSV curve of the Step 1 for H_2 production and the I-V curve of the Si solar cell under simulated AM 1.5 G light illumination.

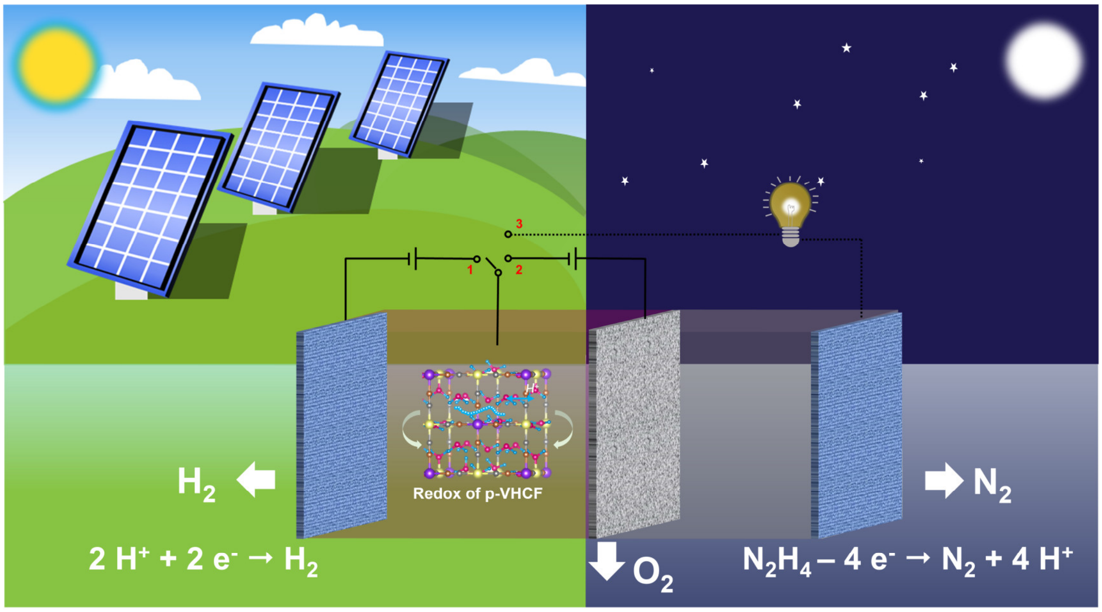

Supplementary Fig. 23 Schematic of flexible energy conversion and storage system by applying decoupled electrolysis.

Supplementary Table 1 Comparison of key metrics of solid-state redox mediators for acid water electrolysis with the literature.

Mediator	Electrolyte	Current density (A g^{-1})	Capacity retention (%)	Cycle number	Capacity (mAh g^{-1})	Ref.
p-VHCF	6 M H_2SO_4	10	100	6000	128	This work
p-VHCF	0.5 M H_2SO_4	10	78.8	6000	95	
CuFe TBA	0.5 M H_2SO_4	1	61	5000	81	1
HATN	0.5 M H_2SO_4	5	51	3000	260	2
MoO_3	10 M H_3PO_4	10	78	500	200	3
NaNiHCF	0.5 M H_2SO_4+ 1 M Na_2SO_4	0.1	92.6	100	48	4
PANI	0.5 M H_2SO_4	0.2	92	40	125	5
PTO	0.5 M H_2SO_4	1	80	200	135	6
PTPAn	0.5 M H_2SO_4	1	76.5	100	63	7

Supplementary References

1. Liang, S., Jiang, M., Luo, H., Ma, Y. and Yang, J. A high-rate electrode with Grotthuss topochemistry for membrane-free decoupled acid water electrolysis. *Adv. Energy Mater.* **11**, 2102057 (2021).
2. Wu, K., Li, H., Liang, S., Ma, Y. and Yang, J. Phenazine-based compound realizing separate hydrogen and oxygen production in electrolytic water splitting. *Angew. Chem. Int. Ed.* **62**, e202303563 (2023).
3. Ma, Z., Lu, X., Park, S., Shinagawa, T., Okubo, M., Takanabe, K. and Yamada, A. High-rate Decoupled water electrolysis system integrated with α-MoO_3 as a redox mediator with fast anhydrous proton kinetics. *Adv. Funct. Mater.* **33**, 2 214466 (2023).
4. Lv, F., Qin, Z., Wu, J., Pan, L., Liu, L., Chen, Y. and Zhao, Y. Decoupled water electrolysis driven by 1 cm^2 single perovskite solar cell yielding a solar-to-hydrogen efficiency of 14.4%. *ChemSusChem*. **16**, e202201689 (2023).
5. Wang, J., Ji, L., Teng, X., Liu, Y., Guo, L. and Chen, Z. Decoupling half-reactions of electrolytic water splitting by integrating a polyaniline electrode. *J. Mater. Chem. A.* **7**, 13149-13153 (2019).
6. Ma, Y., Guo, Z., Dong, X., Wang, Y. and Xia, Y. Organic proton-buffer electrode to separate hydrogen and oxygen evolution in acid water electrolysis. *Angew. Chem. Int. Ed.* **58**, 4622-4626 (2019).
7. Ma, Y., Dong, X., Wang, Y. and Xia, Y. Decoupling hydrogen and oxygen production in acidic water electrolysis using a polytriphenylamine-based battery electrode. *Angew. Chem. Int. Ed.* **57**, 2904-2908 (2018).
