# Supplementary material for: Decoupled electrolysis for hydrogen production and hydrazine oxidation via high-capacity and stable pre-protonated vanadium hexacyanoferrate
Source: Nat Commun. 2024 Feb 13;15:1339. doi: 10.1038/s41467-024-45321-z (PMC10864379; doi:10.1038/s41467-024-45321-z)
Supplement: Supplementary file 3 — Description of additional supplementary files [file 41467_2024_45321_MOESM3_ESM.pdf]

Description of Additional Supplementary Files

Title: Supplementary Movie 1.

Description: Decoupled electrolysis for hydrogen production (Step 1) and oxygen production (Step 2) at 100 mA.

Title: Supplementary Movie 2.

Description: Decoupled electrolysis for hydrogen production at 100 mA (Step 1) and hydrazine oxidation at 10 mA (Step 2’).
